# Effect of Human Chorionic Gonadotropin Injection Before Frozen Embryo Transfer on Pregnancy Outcomes in Endometriosis-Associated Infertility

**DOI:** 10.3389/fmed.2020.592921

**Published:** 2020-12-14

**Authors:** Yanbo Du, Lei Yan, Mei Sun, Yan Sheng, Xiufang Li, Zhenhua Feng, Rong Tang

**Affiliations:** ^1^Reproductive Hospital Affiliated to Shandong University, Jinan, China; ^2^School of Medicine, Shandong University, Jinan, China; ^3^National Research Center for Assisted Reproductive Technology and Reproductive Genetics, Key Laboratory of Reproductive Endocrinology, Shandong University, Ministry of Education, Jinan, China; ^4^Shandong Provincial Hospital, Jinan, China

**Keywords:** endometriosis, frozen embryo transfer, human chorionic gonadotropin, hormone replacement, assisted reproductive technology

## Abstract

**Purpose:** The aim of this study was to investigate the effect of human chorionic gonadotropin (hCG) in hormone replacement (HT) regime for frozen thawed embryo transfer in women with endometriosis (EM).

**Methods:** We performed a retrospective, database-search, cohort study and included data on EM patients who underwent frozen embryo transfer (FET) between January 1, 2009 and August 31, 2018. According to the protocols for FET cycle, the patients were divided into two groups: control group (*n* = 296) and hCG group (*n* = 355). Clinical pregnancy rate, live birth rate, early abortion rate, late abortion rate, and ectopic pregnancy rate were compared between the two groups.

**Results:** There was a significant increase in clinical pregnancy rate in the hCG group (57.7 vs. 49%, *p* = 0.027) compared with the control group. The live birth rate in the hCG group (45.6 vs. 38.5%, *p* = 0.080) was also elevated, but this difference was not statistically significant.

**Conclusion:** hCG administration in HT regime for FET increases the pregnancy rate in women with EM.

## Introduction

Endometriosis (EM) is a chronic gynecological disease characterized by lesions of endometrial-like tissue outside of the uterine cavity (1, 2). Almost 50% of women with EM experience infertility (3). EM affects the outcomes of assisted reproductive technology (ART). There are different factors involved in poor outcomes, including poor quality of oocyte and/or embryo, impaired receptivity of the endometrium, and implantation failure (4, 5). Clinically, there is little doubt that the endometrium of women with EM is less receptive to embryo implantation, and strong evidence suggests that endometrial changes are associated with decreased cycle fecundity (6). Endometrial biomarkers are differentially expressed in the endometrium of women with EM compared with normal women (7, 8). Seeking an effective approach to improve endometrial receptivity in EM is a complex clinical issue.

Frozen embryo transfer (FET) is recommended for EM in ART. Mohamed et al. suggested that the preparation of the endometrium for FET with gonadotropin-releasing-hormone (GnRH) agonists could improve the live birth rate in EM compared with FET (9). Recently, Xu et al. found that pregnancy rate, clinical pregnancy rate, and birth weight were improved in women with EM who underwent intrauterine injection of human chorionic gonadotropin (hCG) before FET (10). However, these methods are either time-consuming or inconvenient. Therefore, we retrospectively analyzed the data on FET in patients with EM to determine a more effective strategy for FET. The aim of our study was to investigate the effect of hCG injection in hormone replacement (HT) regime on FET outcomes among patients with EM.

## Materials and Methods

### Subjects

This retrospective, database-search, cohort study was conducted at the Reproductive Hospital Affiliated Shandong University, Shandong, China. This study was reviewed and approved by the research ethics committee of our institution. All included patients provided written informed consent for use of their data.

All patients who were diagnosed with laparoscopically confirmed EM and underwent FET in our hospital during a 9-year period (January 1, 2009 to August 31, 2018) were included in the study. However, the stage of EM was not clear, as the operation records were not provided. Patients older than 42 years at the onset of the cycle; with basal follicle stimulating hormone (FSH) level >12 U/L; and with uterine malformation, chromosomal abnormalities, hydrosalpinx, recurrent spontaneous abortion, and submucosal fibroid or intrauterine adhesions were all excluded from the study. Finally, data on 651 women diagnosed with EM-associated infertility were collected. According to whether hCG injection was included in the protocols, the patients were divided into two groups—control group (*n* = 296) and hCG group (*n* = 355).

### Endometrial Preparation

Patients in the control group received HT regime for FET. Oral estradiol valerate (Progynova, Delpharm Lille) at a dose of 4–8 mg/day was initiated on day 2 or 3 of the menstrual cycle and commonly lasted for 10–14 days to promote endometrial proliferation. The dosage and duration of estrogen were increased until the endometrial thickness reached an appropriate state for embryo transfer (commonly, at least 8 mm), at which time vaginal progestin (200 mg/day Utrogestan, Besins Healthcare) and oral dydrogesterone (20 mg b.i.d Duphaston, Abbott) were added. Patients who received FET earlier than 2011 were treated with intramuscular injection of progesterone (Progesterone Injection, Zhejiang Xianju Pharmaceutical Co., Ltd) instead.

### Embryo Transfer

According to the embryo morphology assessment by the Istanbul consensus workshop, blastocysts on day 5 with inner cell mass in the hatching stage and trophectoderm layer (grade 1–2) were considered good embryos. While, as cleavage stage embryos, high-quality embryos were scored by morphologic criteria (11). Only good embryos were frozen and prepared for transfer. One or two cleavage-stage embryos or blastocysts frozen early were thawed and transferred 3 or 5 days, respectively, after progesterone initiation. Women in the hCG group received 8,000 IU hCG (HCG; Livzon) by intramuscular injection before progestin administration; the HT regime was the same as the control group. If conception was confirmed, estrogen was continued until 7 weeks of gestation, and progestin supplementation was continued until 10 weeks of gestation.

### Outcomes

The primary outcome was clinical pregnancy, which was defined as the detection of an intrauterine gestational sac by transvaginal ultrasonography after 3 weeks of FET. The secondary outcome was live birth, which was defined as the delivery of a viable neonate that was at least of 28 gestational weeks. The early abortion rate was defined as the percentage of miscarriage occurring earlier than 12 weeks, whereas the late abortion rate was the proportion of miscarriage occurring between 12 and 28 weeks. Preterm birth rate was defined as the percentage of birth before 34 weeks in women with live birth. The live birth rate was the proportion of women who birthed at least one living child.

### Statistical Analysis

Continuous variables were represented as means and standard deviations; differences in variables were compared by means of Student's *t*-test. Categorical variables were described as frequencies and percentages, with the between-group difference tested by means of the chi-square test and Fisher's exact test when the number of events was <5. A two-sided *p*-value < 0.05 was considered to indicate statistical significance.

## Results

### Patients

The baseline characteristics were similar in the control and hCG groups (Table 1). There were no differences in terms of age, body mass index (BMI), basal endocrine hormone levels, number of oocytes retrieved in the fresh cycle, fertilization rate of oocytes, number of available embryos, and the percentage of patients with coexisting polycystic ovarian syndrome (PCOS) and adenomyosis between the two groups. However, the level of anti-Müllerian hormone (AMH) was lower in the hCG group than in the control group.

**Table 1:** Basal characteristics of participants.

	**Control group**	**hCG group**	***p*-value**
*N*	296	355	
Age (years)	31.51 ± 4.33	31.39 ± 4.14	0.663
BMI (kg/m^2^)	22.41 ± 3.96	22.92 ± 3.33	0.627
AMH (ng/mL)	5.53 ± 5.38	5.14 ± 4.12	0.003
Concurrent PCOS	50 (16.9%)	49 (13.8%)	0.276
Concurrent adenomyosis	4 (1.4%)	7 (2%)	0.762
Basal FSH (U/L)	6.75 ± 2.97	6.50 ± 1.85	0.096
Basal LH (U/L)	5.89 ± 3.47	5.73 ± 3.29	0.818
Basal E2 (pg/mL)	42.93 ± 32.40	49.33 ± 111.5	0.103
No. of oocyte retrieved	14.44 ± 7.96	13.87 ± 7.31	0.168
Fertilization rate of oocytes	69.4%	65.6%	0.639
No. of available embryos	5.27 ± 3.33	4.75 ± 2.66	0.066

*There were no significant differences between the hCG group and the control group (p > 0.05) in any of the baseline characteristics except AMH*.

### Outcomes of FET

The concentration of estrogen and luteinizing hormone (LH), the number and stage of transferred embryos, and the proportion of patients with recurrent endometrioma did not differ significantly between the two groups, but the endometrium was thicker in the hCG group than in the control group. FET outcomes were improved in the hCG group. The pregnancy rate of the hCG group was significantly higher than that of the control group (57.7 vs. 49%, *p* = 0.027) (Table 2). The live birth rate of the hCG group (45.6 vs. 38.5%, *p* = 0.080) was also higher than that of the control group, but this difference was not statistically significant (Table 2). In addition, there was no significant difference in early abortion rate, late abortion rate, preterm birth rate, and ectopic pregnancy rate between the two groups (Table 2). In a multivariate logistic regression model, we analyzed various clinical factors of the patients, and we found that hCG treatment, the age of patients, and recurrent endometrioma with a diameter >3 cm were associated with the pregnancy rate of FET in EM patients (Table 3).

**Table 2:** Outcomes of frozen embryo transfer.

	**Control group**	**hCG group**	***p*-value**
Endometrium thickness	0.90 ± 0.30	0.92 ± 0.13	0.000
**Recurrent endometrioma**			
Cyst diameter (<3 cm)	64 (21.6%)	72 (20.3%)	0.700
Cyst diameter (≥3 cm)	34 (11.5%)	37 (10.4%)	0.706
Transferred embryo stage			0.425
Cleavage stage	22 (7.2%)	20 (5.5%)	
Blastocyte	283 (92.8%)	342 (94.5%)	
No. of transferred embryos	1.29 ± 0.56	1.33 ± 0.51	0.965
Estrogen	262.91 ± 294.51	293.39 ± 342.17	0.331
LH	18.42 ± 11.66	15.19 ± 13.66	0.296
Pregnancy rate	49.0%	57.7%	0.027^*^
Live birth rate	38.5%	45.6%	0.080
Preterm birth rate	4.4%	11.7%	0.040^*^
Early abortion rate	8.8%	11.0%	0.362
Late abortion rate	0%	0.8%	0.255
Ectopic pregnancy rate	1.4%	0.3%	0.183

* *p < 0.05*,

** *p < 0.01*.

**Table 3:** Multivariable logistic regression analysis.

	**Variables in the equation**				
		***p-*****value**	**OR**	**95.0% CI**	
Step 1a	Group	0.023^*^	0.444	0.221	0.894
	Age	0.030^*^	0.900	0.818	0.990
	AMH	0.486	1.027	0.952	1.109
	Cyst <3 cm	0.241	0.594	0.248	1.419
	Cyst ≥ 3 cm	0.015^*^	3.878	1.306	11.510

*Adjusted by age, BMI, AMH, basal FSH, basal LH, basal E2, the number of transferred embryos, the thickness of the endometrium, the level of estrogen and LH before FET, the number of retrieved oocytes fertility rate, the number of available embryos in fresh cycle, and recurrent endometrioma in the FET process*.

* *p < 0.05*.

## Discussion

In our retrospective analysis, we found that hCG injection could significantly improve pregnancy rate in EM patients who underwent FET. Further, live birth rate was also elevated in the hCG group; however, the difference was not statistically significant.

LH/hCG receptors are detected in the glandular epithelium cells of the human endometrium (12), and hCG has been shown to promote human endometrium stromal cell decidualization when used in combination with progesterone (13). Transcription of hCG was found in two-cell-stage embryos (14), and the specific interaction of blastocyst-derived hCG and the endometrial LH/hCG receptors is a fundamental component in the materno-fetal interface dialogue (15). It has recently been reported that intrauterine hCG could improve FET outcomes for EM patients (10). However, intramuscular injection of hCG, in HT regime in EM patients who underwent FET, has never been reported. We found that hCG intramuscular injection before FET in EM could also improve pregnancy rate, but have no effect on live birth rate. However, the administration of hCG was not beneficial for FET in human replacement cycles for non-EM patients. The main reason for this was assumed to be the distinctive characteristics of EM.

EM is known as an immune disorder and a progesterone-resistant condition, which leads to impaired endometrial receptivity and reduced fertility (16–19). The expression of endometrial biomarkers is altered in the eutopic endometrium of patients with EM compared with normal women.

Lessey et al. reported that endometrial integrins, which are known as cell-surface receptors for extracellular matrix proteins, play an important role in embryo implantation and are decreased in women with infertility and EM (20). Reduced integrin expression is also associated with poor *in vitro* fertilization (IVF) outcomes (21). *In vivo*, hCG could up-regulate integrinβ5 expression in stromal cells from ectopic lesions in EM (22). Other key molecules, such as HOXA10, are required for normal endometrial receptivity (23), and their expression is also reduced in EM. It has been reported that the expression of HOXA10 could be induced by hCG (24). Therefore, hCG injection may improve endometrial receptivity *via* regulating key molecules related to embryo implantation.

Moreover, hCG was also reported to regulate progesterone expression *via* the ERK1/2 pathway (25) and promote human endometrial stromal cell decidualization (13).

Furthermore, an aberrant subset of uterine Natural Killer (uNK) cells was found in the eutopic endometrium of women with EM-associated infertility (26). Immature uNK cell populations exist in infertile women with EM (27), and hCG has been reported as a regulator of uNK cell proliferation, mediated *via* the mannose receptor (CD206) (28). These findings likely suggest that hCG could improve endometrial receptivity *via* regulating immune cells in the eutopic endometrium of women with EM-associated infertility.

The main limitation of our study is that only patients with appropriate endometrial thickness and good quality embryos were included. Furthermore, owing to the retrospective design of our study, the stage of EM could not be provided based on the collected data. Further evidence is needed to validate and determine an optimum protocol with maximum beneficial outcomes.

In our research, the pregnancy rate significantly increased after hCG injection in women with EM; however, the difference in live birth rate was not statistically significant with respect to that of controls. This may be the result of insufficient time or dose of hCG that may be improved by replacing single hCG treatments with a repetitive administration scheme (29).

## Data Availability Statement

The raw data supporting the conclusions of this article will be made available by the authors, without undue reservation.

## Ethics Statement

The studies involving human participants were reviewed and approved by research ethics committee of Reproductive Hospital affiliate d to Shandong University. The patients/participants provided their written informed consent to participate in this study.

## Author Contributions

RT designed the study. YD interpreted the data and drafted the article. LY revised the manuscript critically and accounted for all aspects of the work. MS and YS collected all the data. XL and ZF analyzed the data. All the authors contributed equally to this manuscript.

## Conflict of Interest

The authors declare that the research was conducted in the absence of any commercial or financial relationships that could be construed as a potential conflict of interest.

## References

[1] Senapati S Sammel MD Morse C Barnhart KT. Impact of endometriosis on in vitro fertilization outcomes: an evaluation of the Society for Assisted Reproductive Technologies Database. Fertil Steril. (2016) 106:164–71 e1. 10.1016/j.fertnstert.2016.03.037 27060727 PMC5173290

[2] Johnson NP Hummelshoj L Consensus on current management of endometriosis. Hum Reprod. (2013) 28:1552–68. 10.1093/humrep/det050 23528916

[3] Meuleman C Vandenabeele B Fieuws S Spiessens C Timmerman D D'Hooghe T. High prevalence of endometriosis in infertile women with normal ovulation and normospermic partners. Fertil Steril. (2009) 92:68–74. 10.1016/j.fertnstert.2008.04.056 18684448

[4] Du YB Gao MZ Shi Y Sun ZG Wang J. Endocrine and inflammatory factors and endometriosis-associated infertility in assisted reproduction techniques. Arch Gynecol Obstet. (2013) 287:123–30. 10.1007/s00404-012-2567-0 23053311

[5] Miller JE Ahn SH Monsanto SP Khalaj K Koti M Tayade C. Implications of immune dysfunction on endometriosis associated infertility. Oncotarget. (2017) 8:7138–47. 10.18632/oncotarget.12577 27740937 PMC5351695

[6] Lessey BA Kim JJ. Endometrial receptivity in the eutopic endometrium of women with endometriosis: it is affected, and let me show you why. Fertil Steril. (2017) 108:19–27. 10.1016/j.fertnstert.2017.05.031 28602477 PMC5629018

[7] May KE Villar J Kirtley S Kennedy SH Becker CM. Endometrial alterations in endometriosis: a systematic review of putative biomarkers. Hum Reprod Update. (2011) 17:637–53. 10.1093/humupd/dmr013 21672902

[8] Aghajanova L Velarde MC Giudice LC. Altered gene expression profiling in endometrium: evidence for progesterone resistance. Semin Reprod Med. (2010) 28:51–8. 10.1055/s-0029-1242994 20104428

[9] Mohamed AM Chouliaras S Jones CJ Nardo LG. Live birth rate in fresh and frozen embryo transfer cycles in women with endometriosis. Eur J Obstet Gynecol Reprod Biol. (2011) 156:177–80. 10.1016/j.ejogrb.2011.01.020 21353737

[10] Xu Z Chen W Chen C Xiao Y Chen X. Effect of intrauterine injection of human chorionic gonadotropin before frozen-thawed embryo transfer on pregnancy outcomes in women with endometriosis. J Int Med Res. (2019) 47:2873–80. 10.1177/0300060519848928 31119991 PMC6683888

[11] Puissant F Van Rysselberge M Barlow P Deweze J Leroy F. Embryo scoring as a prognostic tool in IVF treatment. Hum Reprod. (1987) 2:705–8. 10.1093/oxfordjournals.humrep.a136618 3437050

[12] Reshef E Lei ZM Rao CV Pridham DD Chegini N Luborsky JL. The presence of gonadotropin receptors in nonpregnant human uterus, human placenta, fetal membranes, and decidua. J Clin Endocrinol Metab. (1990) 70:421–30. 10.1210/jcem-70-2-421 1688865

[13] Koch Y Wimberger P Grummer R. Human chorionic gonadotropin induces decidualization of ectopic human endometrium more effectively than forskolin in an in-vivo endometriosis model. Exp Biol Med (Maywood). (2018) 243:953–62. 10.1177/1535370218782658 29886768 PMC6108049

[14] Jurisicova A Antenos M Kapasi K Meriano J Casper RF. Variability in the expression of trophectodermal markers beta-human chorionic gonadotrophin, human leukocyte antigen-G and pregnancy specific beta-1 glycoprotein by the human blastocyst. Hum Reprod. (1999) 14:1852–8. 10.1093/humrep/14.7.1852 10402404

[15] Perrier d'Hauterive S Berndt S Tsampalas M Charlet-Renard C Dubois M Bourgain C. Dialogue between blastocyst hCG and endometrial LH/hCG receptor: which role in implantation? Gynecol Obstet Invest. (2007) 64:156–60. 10.1159/000101740 17934312

[16] Braundmeier A Jackson K Hastings J Koehler J Nowak R Fazleabas A. Induction of endometriosis alters the peripheral and endometrial regulatory T cell population in the non-human primate. Hum Reprod. (2012) 27:1712–22. 10.1093/humrep/des083 22442246 PMC3357193

[17] Berbic M Hey-Cunningham AJ Ng C Tokushige N Ganewatta S Markham R. The role of Foxp3+ regulatory T-cells in endometriosis: a potential controlling mechanism for a complex, chronic immunological condition. Hum Reprod. (2010) 25:900–7. 10.1093/humrep/deq020 20150173

[18] Bulun SE Cheng YH Yin P Imir G Utsunomiya H Attar E. Progesterone resistance in endometriosis: link to failure to metabolize estradiol. Mol Cell Endocrinol. (2006) 248:94–103. 10.1016/j.mce.2005.11.041 16406281

[19] Wolfler MM Kuppers M Rath W Buck VU Meinhold-Heerlein I Classen-Linke I. Altered expression of progesterone receptor isoforms A and B in human eutopic endometrium in endometriosis patients. Ann Anat. (2016) 206:1–6. 10.1016/j.aanat.2016.03.004 27050108

[20] Lessey BA Castelbaum AJ Sawin SW Buck CA Schinnar R Bilker W. Aberrant integrin expression in the endometrium of women with endometriosis. J Clin Endocrinol Metab. (1994) 79:643–9. 10.1210/jcem.79.2.7519194 7519194

[21] Miller PB Parnell BA Bushnell G Tallman N Forstein DA Higdon HL III. Endometrial receptivity defects during IVF cycles with and without letrozole. Hum Reprod. (2012) 27:881–8. 10.1093/humrep/der452 22246449 PMC3279128

[22] Huber A Hudelist G Knofler M Saleh L Huber JC Singer CF. Effect of highly purified human chorionic gonadotropin preparations on the gene expression signature of stromal cells derived from endometriotic lesions: potential mechanisms for the therapeutic effect of human chorionic gonadotropin in vivo. Fertil Steril. (2007) 88:1232–9. 10.1016/j.fertnstert.2007.02.006 17561010

[23] Du H Taylor HS. The role of hox genes in female reproductive tract development, adult function, and fertility. Cold Spring Harb Perspect Med. (2015) 6:a023002. 10.1101/cshperspect.a023002 26552702 PMC4691806

[24] Fogle RH Li A Paulson RJ. Modulation of HOXA10 and other markers of endometrial receptivity by age and human chorionic gonadotropin in an endometrial explant model. Fertil Steril. (2010) 93:1255–9. 10.1016/j.fertnstert.2008.11.002 19131054

[25] Tapia-Pizarro A Archiles S Argandona F Valencia C Zavaleta K Cecilia Johnson M. hCG activates Epac-Erk1/2 signaling regulating Progesterone Receptor expression and function in human endometrial stromal cells. Mol Hum Reprod. (2017) 23:393–405. 10.1093/molehr/gax015 28333280

[26] Giuliani E Parkin KL Lessey BA Young SL Fazleabas AT Characterization of uterine NK cells in women with infertility or recurrent pregnancy loss and associated endometriosis. Am J Reprod Immunol. (2014) 72:262–9. 10.1111/aji.12259 24807109 PMC4126872

[27] Thiruchelvam U Wingfield M O'Farrelly C. Increased uNK progenitor cells in women with endometriosis and infertility are associated with low levels of endometrial stem cell factor. Am J Reprod Immunol. (2016) 75:493–502. 10.1111/aji.12486 26791471

[28] Kane N Kelly R Saunders PT Critchley HO. Proliferation of uterine natural killer cells is induced by human chorionic gonadotropin and mediated via the mannose receptor. Endocrinology. (2009) 150:2882–8. 10.1210/en.2008-1309 19196802 PMC2709965

[29] Makrigiannakis A Vrekoussis T Zoumakis E Kalantaridou SN Jeschke U. The role of HCG in implantation: a mini-review of molecular and clinical evidence. Int J Mol Sci. (2017) 18:1305. 10.3390/ijms18061305 28629172 PMC5486126

[30] Du Y Yan L Sun M Sheng Y Li X Feng Z Effect of human chorionic gonadotropin injection before frozen embryo transfer on pregnancy outcomes in endometriosis infertility. Research Square [preprint]. (2020). 10.21203/rs.3.rs-15724/v1 PMC7768008 33381512

