# Supplementary material for: Effect of Human Chorionic Gonadotropin Injection Before Frozen Embryo Transfer on Pregnancy Outcomes in Endometriosis-Associated Infertility
Source: Front Med (Lausanne). 2020 Dec 14;7:592921. doi: 10.3389/fmed.2020.592921 (PMC7768008; doi:10.3389/fmed.2020.592921)
Supplement: Supplementary file 1 [file Data_Sheet_1.PDF]

Supplementary

S-table 1. Sub-group analysis according to age

Age	≤35 years old			＞35years old		
Groups	Control group<br>(n=238)	HCG group<br>(n=290)	p value	Control group<br>(n=58)	HCG group<br>(n=65)	p value
Pregnancy rate	48.3%	62.2%	0.002*	51.7%	38.5%	0.151
Live birth rate	38.2%	49.3%	0.014*	39.7%	29.2%	0.256

* means p<0.05. HCG could significantly increase pregnancy rate and live birth rate in EM patients less than 35 years old, while HCG had no effect on outcomes of FET in endometriosis more than 35 years old.

S-table 2. Basal characteristics of patients less 35 years old

	Control group	HCG group	p value
N	238	290	
Age(Years)	29.89±2.97	29.96±3.04	0.571
BMI(kg/m^2)	22.91±2.97	22.86±3.32	0.627
AMH(ng/ml)	5.09±6.12	4.73±4.42	0.091
Basal FSH(U/L)	6.56±3.04	6.32±1.78	0.313
Basal LH(U/L)	5.87±3.30	5.90±3.49	0.454
Basal E2(pg/ml)	43.07±34.72	52.26±122.5	0.090
No. of oocyte retrieved	15.51±7.70	14.64±7.44	0.346

Fertility rate of oocytes	68.5%	63.9%	0.833
No. of available embryos	5.49±3.01	4.89±2.76	0.053
Endometrium	0.91±0.31	0.92±0.12	0.000*

* means p<0.05

S-table 3. Basal characteristics of patients more than 35 years old

	Control group	HCG group	p value
N	58	65	
Age(Years)	38.16±2.12	37.78±1.60	0.010*
BMI(kg/m^2)	22.15±2.80	22.81±2.43	0.298
AMH(ng/ml)	3.50±2.74	3.11±2.06	0.007*
Basal FSH(U/L)	7.53±2.54	7.35±1.93	0.027*
Basal LH(U/L)	5.96±4.10	5.00±1.99	0.016*
Basal E2(pg/ml)	42.36±20.98	35.95±25,15	0.607
No. of oocyte retrieved	10.00±7.55	10.42±5.53	0.355
Fertility rate of oocytes	72.79%	73.38%	0.626
No. of available embryos	4.41±4.33	4.14±2.10	0.056
Endometrium	0.89±0.11	0.91±0.15	0.367

* means p<0.05
